# Porcelain Inlays as Made by Dr. Jenkins, of Dresden

**Published:** 1899-02

**Authors:** Edwin T. Darby

**Affiliations:** Philadelphia


### International Dental Journal.

Vol. XX. February, 1899. No. 2.

### Original Communications.[1]

### PORCELAIN INLAYS AS MADE BY DR. JENKINS, OF DRESDEN.[2]

BY EDWIN T. DARBY, M.D., D.D.S., PHILADELPHIA.

Mr. President and Members of the Academy,—I almost feel like apologizing for appearing before you without a paper, and yet you will understand that within the limited period given me by the council, when it asked me to prepare one, I could not do the subject justice, so I will ask your indulgence while I speak to you upon the subject of "Porcelain Inlays as made by Dr. Jenkins, of Dresden."

I think all of you have felt the need of something in the way of porcelain inlays. We have not had, up to the present time, what we have needed in that line. There are a great many cases that present themselves where we would be glad to have something better than gold in the anterior part of the mouth; that almost goes without saying. You and I have felt that we would be glad to have something better for labial surfaces, and for exposed proximal surfaces. Gold is unsightly, and just in proportion as people are

[1] The editor and publishers are not responsible for the views of authors of papers published in this department, nor for any claim to novelty, or otherwise, that may be made by them. No papers will be received for this department that have appeared in any other journal published in the country.

[2] Reported stenographically from an extempore lecture delivered before the Academy of Stomatology of Philadelphia.

refined in their tastes they dislike the appearance of this in the anterior part of the mouth. The day has passed for gold fillings to be called beautiful, and the highest degree of skill is shown in concealing rather than exposing our operations. The experience we have had with porcelain inlays has been unsatisfactory. The old method of taking a bit or fragment of a tooth (Ash & Sons or our American teeth) and fitting it to the cavity is a tedious operation, attended with the loss of a great deal of time, and has not given satisfactory results. Good operations can be done in that way, but it requires such painstaking efforts and such an enormous amount of time that we shrink from the operation. The system that Dr. Howe invented some years ago, of baking porcelain rods and making all cavities round, adapting these pieces to cavities, has also been unsatisfactory. In many cases a cavity can, without detriment to a tooth, be made round. Congenital defects, frequently found in the incisors, pits, and depressions, can be made round, the inlay adapted to the cavity, and made to look very nice, provided the shade can be matched. Mr. Doll, of England, did more in the way of porcelain inlays than any who preceded him. He introduced a similar system to Dr. Howe, and I might say a well-adapted system of porcelain inlays. He not only makes them round, but elliptical and crescent-shaped, but unfortunately the cavity has to be shaped, in a measure, to fit the inlay. You can, it is true, grind them to suit the cavity, but there is usually a greater loss of tooth-structure than we are willing to make in order to adapt these inlays.

The system that Dr. Jenkins has invented and practises is not altogether new; it was suggested to him by the glass inlays quite prevalent some years ago. I doubt not that many of you have tried the glass inlays and found them so defective that you abandoned them. I think the experience of most men who have had anything to do with these has been such as to make them regret ever having done anything with them. Dr. Jenkins's system partakes somewhat of that method. His facilities for obtaining a knowledge of glass and porcelain work have been very great. Living, as he does, in Dresden, surrounded by the potteries and glass-works of Saxony and Bohemia, he has learned from the potters and glass-workers a great many things that he might not have learned had he been in this country. You may be interested to know something of the man himself, because we all have our ideas of men, even if we have never seen them. Some of you may know Dr. Jenkins personally. A more charming man you have never met; an American by birth, a gentleman of culture and refinement. He went to Germany more than thirty years ago and established himself in Dresden. He has made a great success as a practitioner; he numbers among his *clientèle* some of the most important personages of Germany, and he ranks among the best American dentists of Europe. As a companion, a comrade, no man is superior to him; he is one of the most genial, pleasant, lovable men it has ever been my privilege to meet.

And now for his work. Dr. Jenkins found that the glass inlays as used by Herbst and others in Germany, and as we have used them in America, were a great disappointment to him, and he sought to produce a porcelain, fusing at a comparatively low temperature, which would take the place of glass inlays. He went from pottery to pottery and factory to factory, and gained what information he could from those who were practical workers in porcelain and glass. He found he could make a porcelain that would fuse at a comparatively low temperature, or below the melting-point of pure gold. It was my privilege to spend a week or more with him a year ago last summer, and I saw him do a large number of these operations, and was charmed with the results he produced. I have seen in the mouths of my patients operations that he did two or three years ago; some I have seen since I returned, and they look beautiful, showing no change. Some have objected to his system because the inlay is held in place by zinc phosphate cement. It is true this would seem to be a serious objection, for we all know that these zinc phosphate cements are destructible in the secretions of the mouth; they do disintegrate and wear away; but, so far as my observation has gone, there has been no wasting around these inlays, even in cases of two or three years' standing. You must bear in mind that there is a very thin layer of cement exposed to the secretions of the mouth.

And now I will endeavor to describe, in as few words as possible, the method of making these inlays. It must not be supposed that without some practice perfect results can be obtained. If you undertake it, it would be well to make a few and insert them in extracted teeth before attempting to do them for the mouth. A little practice will enable you to get correct contours and perfect adaptations to the cavity. It is not the kind of work that careless, inartistic men should undertake, but in the hands of men who are the opposite of this, beautiful and satisfactory results may be obtained.

Let us take, for instance, an approximal cavity in an incisor, such as has been prepared in this plaster tooth. You will observe that this cavity has much the shape of the bowl of a spoon. From this cavity the mould can be easily removed. The shaping of the cavity can best be done with a spoon-shaped excavator and round burs. When all decay has been removed and the margins well defined, a piece of No. 30 rolled gold, rather larger than the approximal face of the tooth, is carried well beyond the cervical border of the cavity, and then a pellet of punk or bibulous paper is used for pressing it into the cavity. I have found that I can best keep the gold well up to the cervix by folding the upper edge upon itself and then slipping a piece of floss silk between the folds and wrapping the floss once or more around the neck of the tooth. When the gold has been made to conform to every portion of the cavity, the floss is cut near the tooth, and the loose end pulled away. If this is done with care, the matrix will not be displaced. The matrix is then gently removed from the cavity and placed in an investment of pulverized asbestos, which may be mixed with alcohol or water. You will observe this little platinum cup with a long handle and cap of platinum to cover the invested mould. The investment is made in this little cup because the porcelain is baked in this. After the investment has been slowly dried over the flame of a lamp or gas-burner, the porcelain powder of the desired shade is mixed with absolute alcohol into a thin paste and the mould partially filled. You will observe this little oven in which it is baked is lined with asbestos felt, and upon its under surface an opening for the gas flame from the blow-pipe. You will also observe the manner in which this blow-pipe is mounted upon these little tripods: these act as a stand for the blow-pipe, and save the necessity of holding the pipe in one hand during the process of fusing. The air is furnished by this ingenious little bellows, which is neat and compact. The baking process is not difficult, although considerable heat is required to fuse the porcelain. As there is some shrinkage in the baking of porcelain, it will be necessary to add porcelain powder two and often three or four times, depending somewhat upon the skill of the operator and the size and character of the inlay to be made.

My own experience has been that about three bakings are required for most pieces. There seems to be greater danger in getting too much rather than too little material in the finished inlay. If too full, and the porcelain overlaps the margins of the cavity, it often means a new inlay, beginning at the first step in the procedure.

After the inlay has been fused it should be allowed to cool down slowly; then the gold-foil which formed the matrix should be peeled off, and the inlay tried in the cavity to see that it is an exact fit. If it be found correct, the next step is to cut with a small diamond disk slight grooves around the inlays, that the cement may have additional hold. When this has been done the cavity should next be made retentive in shape. If the cavity be an approximal one, a slight groove at the cervical border and a small pit near the cutting edge will suffice for an incisor; or if a bicuspid or molar, somewhat the same shape should be given the cavity that would be required if a filling of gold or alloy were to be used.

In the selection of a cement the operator should take one which harmonizes with the color of the tooth and inlay. If a darker cement is used the line of junction between the tooth and inlay will be more marked. My own experience has taught me that the Harvard cement is best suited to this kind of work; it seems to last better than any which I have used. In setting the inlay it is well to mix the cement moderately thin, and put a little in the cavity and a small quantity on the under surface of the inlay, and then with a piece of orange-wood, shaped like a wedge, force it between the adjoining tooth and the inlay. A piece of thin linen tape is often useful in drawing the inlay into place, or even a piece of floss silk wound around the tooth two or three times and tied will insure good adaptation. The cement should be allowed to harden for fifteen or twenty minutes before the saliva comes in contact with it. The greatest difficulty which I have encountered has been in restoring the corners of teeth and getting proper contours, and I fancy others who adopt this kind of work will meet with the same difficulty; and yet a little experience will enable us to overcome this. When one has succeeded in producing a good inlay the result is so satisfactory that he feels encouraged to persevere until the most difficult cases have been accomplished.

Dr. Deems, of Baltimore, is with us this evening, and as he has spent considerable time with Dr. Jenkins and has made many of these inlays, I hope he will tell you his experience with this style of work. I have seen some that he has made, and I feel sure that he will be able to tell you some things which I may have omitted, and possibly show you better than I can how these inlays are made. If the light is sufficient and the flow of gas from this burner ample, I will endeavor to make an inlay at the close of this meeting.

Dr. McQuillen was with me in Dresden, and saw Dr. Jenkins make and insert these porcelain inlays, and as he is present to-night I doubt not he will be pleased to testify to the superior character of the operations he saw performed by Dr. Jenkins and his assistants.

